# BEC Defender: QR Code-Based Methodology for Prevention of Business Email Compromise (BEC) Attacks

**DOI:** 10.3390/s24051676

**Published:** 2024-03-05

**Authors:** Anastasios Papathanasiou, George Liontos, Georgios Paparis, Vasiliki Liagkou, Euripides Glavas

**Affiliations:** 1 Cyber Crime Division, Hellenic Police, 173 Alexandras Avenue, 11522 Athens, Greece; 2 Department of Informatics and Telecommunications, University of Ioannina, Kostaki Artas, 47150 Arta, Greece; eglavas@uoi.gr; 3 Department of Materials Science and Engineering, University of Ioannina, 45110 Ioannina, Greece; gliontos1985@gmail.com; 4 Independent Researcher, 10678 Athens, Greece; gpaparis@gmail.com

**Keywords:** business email compromise (BEC), email security, QR code encryption, cryptography, digital communication security, message authentication code, cybersecurity, MAC address, physical properties, authentication

## Abstract

In an era of ever-evolving and increasingly sophisticated cyber threats, protecting sensitive information from cyberattacks such as business email compromise (BEC) attacks has become a top priority for individuals and enterprises. Existing methods used to counteract the risks linked to BEC attacks frequently prove ineffective because of the continuous development and evolution of these malicious schemes. This research introduces a novel methodology for safeguarding against BEC attacks called the BEC Defender. The methodology implemented in this paper augments the authentication mechanisms within business emails by employing a multi-layered validation process, which includes a MAC address as an identity token, QR code generation, and the integration of timestamps as unique identifiers. The BEC-Defender algorithm was implemented and evaluated in a laboratory environment, exhibiting promising results against BEC attacks by adding an extra layer of authentication.

## 1. Introduction

Email has become an integral part of our daily lives, with over 333.2 billion emails sent and received per day in 2022 worldwide [1]. However, the convenience of email has also led to an increase in cyberattacks, including business email compromise (BEC) attacks. In a BEC attack, an attacker impersonates a legitimate sender to deceive the receiver into sending money or sensitive information.

More specifically, in a typical business email compromise (BEC) scheme, the perpetrators carefully select their target and employ a series of tactics to gather valuable information from open-source intelligence (OSINT) techniques [2] and then construct an elaborated malicious email, often assuming the identity of a trusted entity or source. Within this fraudulent email, the attacker may employ sophisticated social engineering techniques, designed to manipulate and coerce the recipient into taking actions that ultimately benefit the scammer. Alternatively, the email may include malicious payloads, such as viruses concealed in various attachments or deceitful links. These malicious actions serve multiple nefarious purposes. Firstly, they aim to compromise the victim’s communication channels, potentially allowing the scammer to intercept sensitive information. Moreover, the attacker may seek to extract money or valuable data from the unsuspecting victim [3,4]. In essence, BEC attacks represent a multifaceted threat that combines careful target selection, information gathering, persuasive impersonation, and the deployment of harmful software or links to achieve illegal objectives.

These attacks are often successful because they exploit human error, such as trusting an email’s contents without verifying its authenticity. In 2022 alone, BEC attacks resulted in losses of nearly USD 2.7 billion globally, which is an escalation of approximately USD 350 million from the preceding year (2021), and a notable surge of around USD 860 million from the year 2020, according to the FBI statistics report [5].

**Figure 1:**
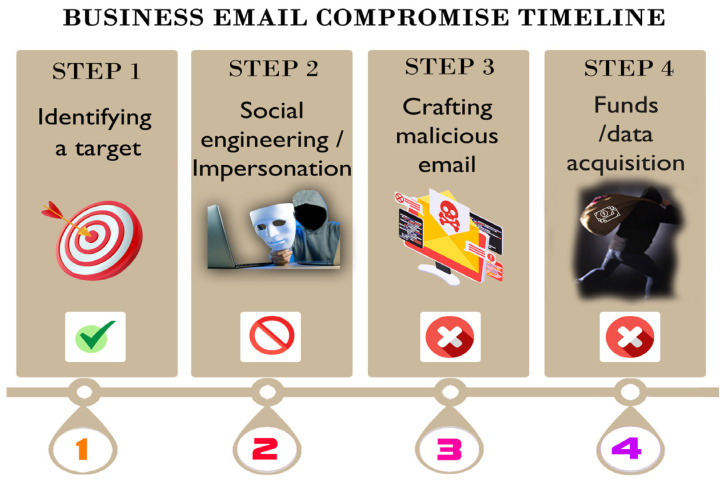
Business email compromise timeline.

Figure 1 depicts a general BEC scheme timeline. In Step 1, the attackers identify a target, most commonly a CEO or CFO. The primary objective of the attacker is to extract financial gains or confidential data by assuming the identity of a high-ranking individual within a corporation. However, the final or intermediary victims can range from the CEO or CFO to employees within different departments, such as accountants or IT personnel. In Step 2, the attackers employ social engineering techniques in order to gather information about the victim or victims (employees or associates in the targeting enterprise), and, in Step 3, the attacker crafts a sophisticated email in order to extract funds or intercept sensitive information (Step 4) [6].

To mitigate business email compromise (BEC) attacks and ensure the security of online communications, individuals, enterprises, and critical infrastructures employ a range of both technical and non-technical tools for their protection.

The primary category of tools, known as technical solutions, encompasses a variety of defenses, such as antivirus software to shield against malicious payloads, antimalware programs, email protocols like DMARC (Domain-Based Message Authentication, Reporting, and Conformance), machine learning algorithms, encryption methods, multi-factor authentication (MFA) solutions, and other specialized services. DMARC shields against domain spoofing through digital signatures, ensuring email integrity, but determined attackers can manipulate email addresses to deceive recipients. Antivirus and antimalware software plays a crucial role in guarding against malicious URLs and programs, relying on widespread adoption and updates, but it remains susceptible to emerging threats and social engineering techniques. Machine learning algorithms show promise in classifying emails and identifying BEC attack patterns but require the analysis of large amounts of data, particularly within email body text. Encryption provides substantial security benefits but faces challenges such as complexity, key management, compatibility issues, and potential processing overhead. Multi-factor authentication (MFA) offers an additional layer of protection but introduces challenges in terms of convenience, compatibility, and the phishing risk, leaving room for uncertainty in BEC attacks.

The second group centers on non-technical safeguards/countermeasures, which are equally vital. Among these, user awareness training stands out as an essential element in educating personnel about BEC threats and phishing tactics. Clearly defined security policies and the establishment of specialized social engineering departments further bolster an organization’s resilience against BEC attacks. The main drawback of non-technical measurements is that they rely heavily on the human factor, introducing unique challenges and demanding constant vigilance and adaptation due to the ever-evolving nature of BEC attacks. In essence, these non-technical strategies are crucial components, but they require a deep understanding of the human element in security, acknowledging its dual role in both bolstering and undermining the defense effectiveness against cybercriminal tactics.

Despite the availability of these multifaceted tools and strategies, the implementation process and the complexity of the defenses can often overwhelm the average user. Experienced scammers, recognizing this challenge, often exploit and capitalize on the vulnerabilities stemming from limited awareness and technical gaps, highlighting the persistent requirement for heightened vigilance, continuous education, and innovative security measures amidst the ever-changing threat environment.

To address this issue, this research proposes a novel methodology called the BEC Defender that leverages various authentication techniques to secure email communication and protect against BEC attacks.

The methodology capitalizes on particular user attributes and QR code technology to secure email communication with an additional layer of authentication. The user attributes encompass the sender’s MAC address, IP address, and hostname, and the email creation timestamp. These attributes are encoded via a dedicated algorithm, resulting in an output that is transmuted into a QR code. Subsequently, the user includes the generated QR code when attaching it to the email, allowing the receiver to authenticate the legitimacy of the sender’s identity. This proposed scheme is designed in its current state for desktop and laptop devices.

With the proposed methodology described in this research, the authors aim to create a tool for defending against BEC attacks by stopping the attacker in Step 2 (Figure 1), which involves the impersonation of a trusted entity or source. The impersonation of a trusted entity is a critical element in business email compromise attacks and significantly contributes to their success.

In this paper, we present the theoretical foundation and implementation details of our solution in an effort to enhance email security and protection against BEC attacks. We also provide an overview of related works and discuss the advantages and limitations of our proposed solution.

## 2. Literature Review Methodology

A systematic search strategy across various academic search engines, such as Google Scholar, Core, Scopus, and Science.gov, were employed to conduct the literature review.

This process involved the utilization of multiple keywords and key phrases, such as “BEC”, “DMARC”, “BEC prevention methods”, “Message Authentication Code–MAC”, “QR technology”, “email security”, etc. Following a pilot search, an inclusion/exclusion procedure was employed in which articles irrelevant to this study were excluded, while those relevant were included and analyzed. Furthermore, additional searches using the referenced works of relevant articles were also conducted (the snowball effect).

**Figure 2:**
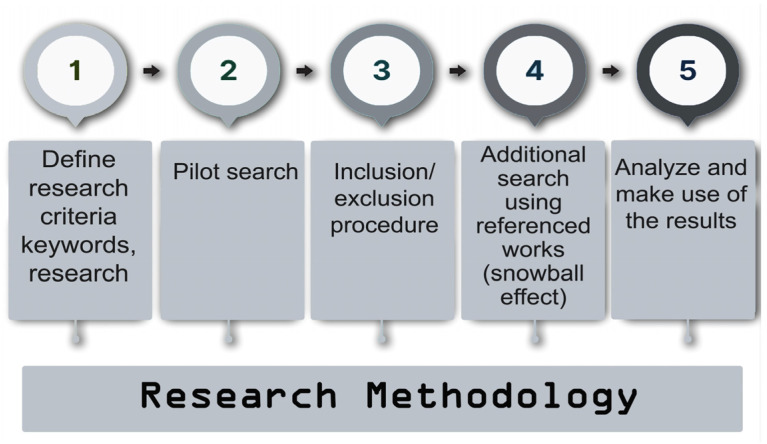
Steps of research methodology.

For the experimental section of this research, various searches were conducted using multiple keywords and key phrases, such as “protection against BEC using physical properties of the computer”, “authentication using MAC address”, “identification using MAC address”, “cryptographic solutions for email protection”, “spoofing MAC addresses”, and others, using the same academic search engines mentioned earlier. The total procedure of the research methodology is depicted in Figure 2.

Despite being utilized in numerous systematic studies, this methodology possesses certain limitations. One limitation is its potential to restrict the scope of the review or study, which could result in readers lacking a comprehensive understanding of the subject matter. Furthermore, our data collection was constrained to only four scientific search engines, potentially limiting the number of publications considered in our review. Although these sources are considered reliable, the limitation lies in not exploring all possible sources to identify relevant articles related to our study objectives.

## 3. Related Work

During our comprehensive literature review, we came across a multitude of innovative approaches aimed at using QR technology in document/data or user authentication. The ensuing paragraphs will clarify several of these solutions, each of which contributes unique tools for authentication and for safeguarding online communication, consequently bolstering the defense against business email compromise (BEC) schemes.

The utilization of QR codes in user or data authentication has been extensively documented in various literature sources, with each source assigning a distinct purpose to QR codes for the verification and authentication of entities or data.

### 3.1. QR Codes in Hard Copies for Document Authentication

According to the available literature, various authentication methods have been explored for validating physical documents using QR codes, as discussed in the subsequent Sections. The incorporation of QR code solutions in these academic works demonstrates promise in the realm of verifying the authenticity of hard-copy documents through the scanning of the QR code by an authentication device. It is worth noting that these methods primarily refer to physical documents and may not be applicable to email communications for countering business email compromise (BEC) attacks.

Singhal A et al. [7] propose a method that verifies a university degree certificate with the use of a QR code that contains a digital signature over the data, such as the degree holder’s name, enrollee number, roll number, etc. To achieve the same objective, Aini Q. et al. [8] propose a method for authenticating a university diploma by integrating blockchain technology patterns within the QR code to verify the certificate. Both of these bibliographic references employ QR codes with the purpose of encoding information in significant documents, such as diplomas and university degrees; however, this method is lacking in terms of confidentiality, which is a necessary tool in counteracting BEC attacks.

Kuacharoen P. et al. [9] propose a method for document verification using QR codes and digital signatures. The process involves composing a message, generating a hash value, and encrypting it with the sender’s private key to create a digital signature. The message and signature are combined, compressed, and stored in a QR code on paper for transmission. Upon receiving the document, the receiver scans the QR code to verify the authenticity. This involves checking the integrity of the information, uncompressing and comparing the hash values, and utilizing Optical Character Recognition (OCR) to further validate the printed message. If all checks pass, the message is confirmed as authentic.

This method provides a secure and efficient means of document verification, combining cryptographic techniques with OCR technology to ensure the integrity and authenticity of printed documents. The process outlined above is intended for document authentication and heavily depends on OCR technology, which is associated with numerous drawbacks, such as formatting issues, constraints related to language and character sets, potential misinterpretations of acronyms and abbreviations, among other limitations. Furthermore, the abovementioned solution is based on a trusted third party, which, in some cases when dealing with BEC attacks, is rendered as a drawback. These factors collectively render it an unreliable solution for ensuring security in online communication.

Tkachenko, I. et al. [10], in their research, introduce a novel QR code variant with dual storage levels, designed specifically for document authentication. This innovative QR code, which the authors named the “two-level QR code”, incorporates both the public and private storage levels. The public level mirrors the standard QR code storage capacity, making it accessible to any conventional QR code reader. In contrast, the private level is created by substituting the black modules with distinct textured patterns and encoding information using q-ary codes with error correction capabilities. This not only enhances the QR code’s storage capacity but also enables the differentiation between the original document and any copies, owing to the sensitivity of these patterns to the print-and-scan (P&S) process. The pattern recognition technique employed to decode the second-level information is versatile, applicable to both private-message-sharing and authentication scenarios. The authentication of the private message is accomplished with ECC-based signatures [11,12]. It relies on the mathematical properties of elliptic curves to provide encryption and decryption capabilities. In ECC, a pair of keys, a public key and a private key, are generated. The public key is used to encrypt the message, while the private key is used to decode (decrypt) it. ECC comes with certain drawbacks, especially when it comes to online communication, such as emails. More specifically, ECC can add complexity to the email security process considered and may not be supported by all email clients and services. Additionally, the abovementioned solution is designed for hard-copy documents, and in order for it to be implemented in online communication systems, factors like replay attacks must be considered thoroughly, especially when it comes to BEC attacks.

### 3.2. QR Codes for Digital Authentication

The literature referenced below discusses innovative approaches to authentication. Most of the references utilize QR codes on trusted devices such as mobile phones or through the involvement of a trusted third party. As groundbreaking as these methods may be, their main drawback is the reliance on trusted devices and third-party involvement.

Lu J. et al. [13] propose a methodology for mobile payment authentication that combines visual cryptography (VCS) and aesthetic QR codes. This approach offers three different levels of concealment. The process involves splitting an original QR code into two shadow versions using VCS rules. These two shadow versions are then separately incorporated into the same background image. The results of this embedding process are combined with an identical carrier QR code using a combination of the Reed–Solomon (RS) XOR mechanism and QR code error correction mechanisms. Finally, the two aesthetically enhanced QR codes can be accurately layered to reveal the original QR code as per the defined visual cryptography scheme. While the described solution focuses on enhancing the security in QR code-based mobile payment authentication by splitting the QR code into shadows and embedding it in a carrier QR code, it does not specifically address the issue of business email compromise (BEC) schemes. Specifically, the above proposed solution does not take into consideration advanced encryption techniques, nor does it address the issue of replay attacks.

Liao K.C. [14] propose a QR code-based, one-time-password authentication protocol, which the author claims eliminates the usage of the password verification table in an improved, cost-effective way. While it shows promise, this project focuses on substituting traditional password authentication methods with QR codes through users’ mobile devices and is unrelated to safeguarding against BEC schemes.

Oh D. S. et al. [15], in order to address the issue of significant network traffic due to frequent user authentication processes in the existing mobile cloud authentication methods, propose an authentication system that optimizes the network traffic usage in mobile cloud environments by implementing QR codes. However, as the authors claim, this method does not analyze the security vulnerabilities of the suggested system in comparison to existing technologies. Furthermore, the proposed solution in this project concentrates on authenticating users in the Public Cloud, also known as a trusted third party, with the aim of aiding small- and medium-sized businesses, but it does not have any relevance to enhancing online communications, particularly in the context of BEC attacks.

Choi K. et al. [16] propose an anti-phishing, single-sign-on (SSO) authentication model using QR codes. In this proposed architecture, an extended authentication server concentrates the user identifier, server information, and random nonce (random key generated by the server) data and encrypts them with a shared secret key. The secret key is shared by a mobile device with extended authentication. In the next step, the extended-authentication server generates a QR code with the abovementioned encrypted data and also a timestamp. Next, the QR code is scanned from a mobile device, which decrypts the data, generates another random nonce (random key generated from the mobile device), again encrypts all the data plus the password, and creates another QR code with the encrypted data. For the verification phase, the mobile device sends the shared data to an authentication server for validation. The user can then compare the user rand displayed on the web server and the user rand displayed on the mobile device in order to confirm the communication. While this highly promising project offers various advantages, it comes with a notable drawback: the use of an extended-authentication server. Although this server’s convenience is apparent, it introduces a potential security risk, as attackers could compromise it. The objective of this project is to improve the user identification and data integrity through the implementation of an identity management system centered around an authentication server. However, this approach may face challenges in its adaptability to business email compromise (BEC) attacks. Instead, token-based identification systems and the use of anonymous credentials might prove more effective. Furthermore, this approach involves the engagement of a trusted third party in the authentication of both the users and data.

Bairwa et al. [17] created an algorithm for message and data transfer using an authentication token containing six-digit random numbers with the SHA-hash parts of the sender’s and receiver’s MAC addresses. In this research, the authors use symmetric-key cryptography and especially message authentication codes (MACs). In order to register to the above program, the user must fill the registration form with their username, email, MAC address, and fingerprint. Next, the program generates a password using SHA-256 algorithms, which develop the hash corresponding to the MAC address and the fingerprint. All these data are stored in the user data table. All the above are essential in order for a session key to be created. The session key is developed using random numbers, the SHA hash of the sender MAC address, and the SHA hash of the destination MAC address. While this is very promising work, the algorithm is designed especially for Mobile Ad Hoc Networks (MANETs) [18]. MANETs offer flexibility and autonomy but come with several disadvantages. One notable drawback is that, due to the dynamic nature of MANETs, maintaining secure and efficient routing becomes challenging, leading to potential routing loops and packet drops. Furthermore, another significant disadvantage is the limited network scalability, as the performance degrades as the number of nodes increases. In general, while showing promise, the methodology mentioned above is complicated and inadequate for use as a viable solution to defend against BEC schemes.

Chen C. [19] proposes a QR code authentication method that includes hidden authentication elements like message authentication codes and cryptographic signatures. The entity generating the QR code can create a concealed QR code using the author’s enrollment process, where these authentication elements are discreetly incorporated into the code. The key advantage of this proposed method is that the QR code’s content remains accessible to standard barcode scanners, and its authenticity can be confirmed offline by authorized users when necessary. In this study, data authentication is achieved through two distinct methods: one involves message authentication codes (MACs), and the other employs digital signatures with asymmetric cryptography. Although the research introduces a novel perspective without the use of trusted devices or external servers, the proposed solution fails to account for certain vulnerabilities, such as replay attacks. Replay attacks and potential man-in-the-middle threats are the reasons why our proposed solution, the BEC Defender, relies on a three-hour timeframe, guaranteed by the time differential between the sender’s and receiver’s timestamps, as a countermeasure against these security risks. Additionally, the BEC Defender utilizes three distinct methods for authentication. These methods consist of a MAC code, authentication of the encrypted sender’s MAC address as a unique identifier, and the time differential between the two timestamps, ensuring a three-hour timeframe. Each of these authentication processes plays a unique and vital role in countering BEC attacks.

Considering the innovative research efforts discussed earlier, in which QR codes and diverse encryption methods were explored for document and user authentication, we sought to integrate some of these techniques while adding others that are not mentioned in the literature. In our innovative project, our objective is to enhance the security of email communication and counteract BEC schemes, trying to provide a comprehensive solution that combines efficiency, safety, and user-friendliness, while also introducing additional authentication measures to further strengthen security (Table 1).

### 3.3. Literature Related to Defense against BEC Attacks

#### 3.3.1. Technical Methods

There are several ways reported in the literature for defending against BEC attacks that include both technical and non-technical methods. As mentioned in our previous work related to BEC schemes and how to countermeasure them [20], the optimum solution is a combination of technical and non-technical measurements, like those mentioned below:

(1) **DMARC**: The DMARC (Domain-Based Message Authentication, Reporting, and Conformance) email authentication protocol enhances security and prevents email spoofing and phishing attacks. DMARC works in correlation with the SPF (Sender Policy Framework) and DKIM (DomainKeys Identified Mail). The SPF is an email authentication method that allows the domain owner to specify which email servers are authorized to send emails on behalf of their domain. It creates a list of authorized sending IP addresses in the domain’s DNS records. When an email is received, the recipient’s email server can check the SPF record to verify whether the sending server is authorized to send emails for that domain. DKIM is another email authentication method that adds a digital signature to outgoing emails. The domain owner generates a unique private key and publishes the corresponding public key in the DNS records. The private key is used to generate a digital signature that is attached to the email header. When the recipient’s email server receives the email, it can retrieve the public key from the DNS records and use it to verify the digital signature. This ensures the sender’s domain authenticity. DMARC builds upon the SPF and DKIM to provide a more comprehensive email authentication framework. It enables the domain owner to define a policy for how the receiving email server should handle emails that fail the SPF or DKIM checks. The DMARC policy can instruct the recipient’s server to either quarantine or reject emails that fail authentication. Additionally, DMARC offers reporting mechanisms that grant domain owners the ability to monitor the usage of their domains for email authentication purposes. It generates comprehensive reports containing details about the emails sent on behalf of the domain, including the outcomes of the SPF and DKIM authentication (whether they passed or failed). These reports play a crucial role in assisting domain owners in detecting any unauthorized utilization of their domains, resolving authentication problems, and obtaining valuable insights into potential phishing attempts [21,22]. In conclusion, DMARC offers protection against domain-spoofing emails, preventing them from reaching users’ inboxes. Through DMARC, it is possible to block, quarantine, and monitor any malicious emails sent from the controlled domain. Numerous email providers, including Google’s Gmail-hosted mailboxes and Microsoft’s Office365, offer support for DMARC policies [23]. Typically, mail-filtering techniques like DMARC are specifically crafted to operate based on the header information within emails. The email-filtering policy is formulated to examine both incoming and outgoing emails, aiming to prevent any suspicious messages originating from deceptive domains. However, this approach exhibits vulnerabilities when it comes to impersonation attacks, wherein emails may originate from domains that fall outside the scope of the filter. Furthermore, the limitation of mail-filtering techniques lies in their exclusive focus on the email header. Consequently, they prove ineffective at safeguarding the email system against certain types of attacks, particularly those rooted in content manipulation. For instance, schemes involving fraudulent invoices, in which the email content itself is manipulated, pose a significant challenge, as the current mail-filtering approach does not extend its protective measures to this aspect of the email composition [24]. As Särökaari [25] also mentions in his thesis, deploying the SPF and DMARC is not enough to prevent sophisticated and targeted phishing attacks. Furthermore, if an attacker is able to gain access to an employee’s email account, having these countermeasures will not provide any protection, as the attacker is in a position to impersonate the compromised user by having access to their email inbox. Moreover, as Särökaari states, the adoption of these technical security control measures has been largely voluntary, with little penalty for noncompliance;
(2) **Antivirus–antimalware software**: BEC attacks rely on careful and sophisticated planning, involving OSINT investigations to gather critical information about the target. The purpose is to establish psychological leverage and gain valuable insights that can be utilized in future fraudulent emails. However, apart from such meticulous approaches, attackers can employ more direct and intrusive techniques, such as utilizing viruses or malware to compromise the victim’s system and extract sensitive data. Key loggers [26] are an example of such malware, recording the victim’s keystrokes and thereby capturing sensitive information, like login credentials, usernames, and passwords. Another example is remote-access tools (RATs) [27], which aim at obtaining unauthorized system access for further exploitation. Moreover, BEC attackers often resort to social engineering tactics, such as sending initial emails containing malicious URLs. These deceptive links mislead the unsuspecting victims into installing the malicious software into their systems or entering fraudulent websites that mimic legitimate platforms, like e-banking sites. Once on these fake websites, victims may unknowingly disclose their confidential information, allowing the attackers to perpetrate identity theft or financial fraud or gain unauthorized access to systems. Given the evolving sophistication of BEC attacks and other forms of cyber threats, antivirus and antimalware software has become an indispensable tool for organizations and individuals to protect themselves from potential harm. These security measures aid in detecting and mitigating various forms of malicious software and deceptive tactics employed by cybercriminals, thereby reducing the risk of falling victim to BEC schemes and similar cybercrimes [28];
(3) **Machine learning algorithms**: Machine learning is a field of study within artificial intelligence that focuses on developing algorithms and models capable of learning from data and making predictions or decisions. When it comes to business email compromise (BEC) attacks, machine learning can be a valuable tool in detecting and preventing such threats. Machine learning can help combat BEC attacks in several ways [29]. Firstly, it can be used to analyze historical email data and identify patterns associated with known BEC attacks. By training machine learning models on such data, they can learn to recognize common characteristics, such as suspicious email addresses, language patterns, or anomalies in email headers. Additionally, machine learning algorithms can be employed to analyze email content and attachments in real time. These algorithms can learn from a variety of features, such as the email’s structure, sender’s reputation, language used, and contextual information. By leveraging these features, the models can identify suspicious emails that exhibit characteristics commonly associated with BEC attacks, such as unexpected changes in account details or urgent requests for funds. Furthermore, machine learning can assist in identifying compromised accounts or unauthorized access attempts. By monitoring the user behavior and detecting deviations from normal patterns, machine learning models can flag potential unauthorized activities, such as login attempts from unfamiliar locations or unusual timeframes. A. Cidon et al. [30] presented BEC-Guard, a detector employed at Barracuda Networks that uses supervised learning to stop business email compromise threats in real time. BEC-Guard detects attacks by using supervised learning algorithms that are trained on an email database that contains millions of emails. These algorithms analyze the header of the email and search for suspicious phrases and links in the email body. Furthermore, BEC-Guard makes use of the public APIs provided by cloud email providers to automatically acquire knowledge about the past communication patterns of each organization. It also employs these APIs to promptly isolate and quarantine emails in real time. According to the writers, BEC-Guard was evaluated using a commercial dataset comprising over 4000 attacks, achieving a precision of 98.2% and a false-positive rate of less than one in five million emails. A drawback of this methodology is the need to continuously train the algorithm due to the continuous evolution of BEC schemes.
  Furthermore, Cohen et al. [31] present a technique for identifying malicious emails through the utilization of machine learning methodologies. By extracting features from complete emails, including the header, body, and attachments, and employing a Random Forest classifier, the approach asserts an impressive accuracy level of 92.9%, with true-positive and false-positive rates standing at 94.7% and 3%, respectively. The dataset used in the performance evaluation was a collection of 33,142 emails (20,307 benign and 12,835 malicious emails) collected between 2013 and 2016. The malicious emails were labeled as such by at least five different antivirus engines using VirusTotal;
(4) **Encryption**: Encryption serves as an effective measure to prevent data breaches by necessitating a pair of cryptographic keys for both the sender and receiver [32]. For example, in identity-based encryption (IBE), the user’s email address functions as the public key, and a centralized entity referred to as the Private Key Generator (PKG) is responsible for generating private keys. Following a preliminary authentication process, the private keys are securely transmitted from the PKG to end users through a secure channel. It is worth noting that identity-based encryption (IBE) schemes are susceptible to the key theft problem, enabling the PKG to decrypt any message [32].
  Emails can also be safeguarded through various plugins [33]. Mailvelope [34] employs manual key management, requiring users to distribute and handle keys manually, which impacts the usability, especially for novice users. Plugins such as Jumble Mail and Secure Gmail [35] rely on PGP and encrypt messages using their managed keys, requiring end users to trust the provider. Routi et al. [36] conducted a study on PGP with the Mailvelope plugin. Despite considerable enhancements in Mailvelope security, its usability remains low and proves challenging for common users lacking knowledge of public-key cryptography. Other solutions, like Private Webmail, Virtu, and Xmail, generate and distribute encryption keys on their servers while concealing the key management process. However, these solutions are paid, and the unavailability of source codes raises trust concerns for end users;
(5) **Multi-factor authentication (MFA)**: MFA offers a robust method of authentication, demanding two or more verification factors to grant access to a resource [37,38];
(6) **Trusted Third Parties (TTPs)**: Ensuring the secure distribution of public keys to the correct parties can pose significant challenges. The trust placed in the public-key infrastructure (PKI) is of the utmost importance. A trusted third party is often needed to facilitate the provision of public- and private-key pairs. The entire security of the system relies on this trusted entity. Any compromise, whether from external attacks (like server code modification) or internal vulnerabilities, has the potential to undermine the security of the entire system. Consequently, organizations may harbor doubts regarding the trustworthiness of the third party responsible for issuing keys or credentials. Concerns may arise regarding the security practices of the provider, their adherence to regulatory compliance, and their ability to withstand external pressures that could jeopardize the integrity of the key management process. Organizations may hesitate to place their trust in a third-party key management center that fails to demonstrate adherence to relevant standards and best practices. Additionally, concerns may arise regarding the location of the key management center and its compliance with data sovereignty requirements. Certain regulations mandate that specific data must remain within defined geographical boundaries, and relying on a third-party provider may raise questions about data jurisdiction. The utilization of PKI methods usually requires that organizations entrust an external entity for building secure communication between users, thereby relinquishing a certain degree of control over the cryptographic keys. Some organizations may be reluctant to surrender this control, particularly when dealing with highly sensitive information. Depending solely on a specific key management provider may result in vendor lock-in, making it difficult and costly to switch to an alternative provider. Organizations may have reservations about relying solely on a single provider for a crucial security function. Additionally, the costs associated with utilizing a key management center can be a determining factor, and organizations require assurance that they can conduct audits and verify the key management processes to ensure compliance and security. The lack of transparency from the key management center can pose a significant obstacle in establishing trust. This involvement of TTPs underscores their significance in fostering secure, fair, and trustworthy email interactions, making them valuable components in the architecture of communication systems. According to Kupcu [39], due to the fact that numerous systems depend on trusted third parties (TTPs) for assurances in fairness, security, and efficiency, there is a critical necessity to decentralize the trust placed in these central entities. Moreover, Paulin et al. [40] state that current service providers offer limited solutions dependent on a trusted third party, hindering their applicability across borders, especially in transnational unions such as the EU. The authors introduce a functional certified email system that achieves the fair non-repudiation of receipt without relying on a trusted third party. The proposed protocol involves encrypting a message and splitting it into a chain of parts, with the recipient gradually acquiring each part and generating proofs-of-receipt for the individual segments. This protocol cryptographically prevents the addressee from obtaining the message in case they terminate the protocol prematurely. The universality of the presented system makes it feasible for unobtrusive operation using existing user agents and email providers. Sabir et al. [33] mention that, in contrast to other applications, like social media, email accounts inherently contain more sensitive data, making a hacked email account a potential source of personal information leakage and unauthorized access to various online services. Moreover, despite users relying on service providers for email privacy, this trust is often exploited for targeted advertisements. Additionally, the risk of attackers targeting and compromising numerous email accounts underscores the vulnerability of email systems, especially when considering the danger of an attack on the internal server itself. For the abovementioned reasons, the authors devised a solution using a PKI (public-key infrastructure) similar to that of Proton Mail [41] with the following objectives. Firstly, the system aims to ensure complete end-to-end privacy. Secondly, it strives for significant usability aligned with the Saltzer acceptability principle, aiming to enable users without technical expertise to navigate the system effortlessly, including aspects such as obtaining, distributing, and utilizing cryptographic keys. Thirdly, portability is emphasized, allowing users to switch between public computers without reliance on a specific device. Fourthly, users are not required to install additional hardware or software configurations to use the system. Lastly, the trustworthiness of the application code is highlighted, emphasizing a transparent, cryptographic key-sharing mechanism to instill user confidence.
  An interesting work is that of AlSabah et al. [42], which presents a secure end-to-end email communication approach. By employing their innovative certificate-less (CL) key agreement protocol, the method enables users to update their public keys without requiring interaction with the certificate authority (CA).
  Moreover, Brown et al. [43] introduced a proxy-based architecture. Proxy-based methods utilize their servers for encrypting and decrypting messages, making them not genuinely end-to-end secure. Jammalamadaka et al. [44] proposed a proxy-based design that necessitates additional hardware (a mobile phone) to execute secure email operations. Another Windows-based system, Opaque-Mail, communicates with mail clients and requires local installation on all users’ devices. Additionally, proxy re-encryption, by design, has an insignificant impact on email privacy. Moreover, user trust could be manipulated by introducing backdoors through application source codes.
  Finally, Secure/Multipurpose Internet Mail Extensions (S/MIMEs) serve as an encryption standard akin to PGP, ensuring the security of email content. Built on public-key cryptography, S/MIMEs mandate the involvement of certificate authorities (CAs) in issuing certificates for both the sender and receiver. This approach necessitates mutual trust in the CA. Despite some companies opting for self-issued certificates, these are often perceived as untrustworthy, potentially introducing security vulnerabilities. Additionally, S/MIMEs fall short in safeguarding users against Vendor Email Compromise (VEC) attacks, particularly when utilizing servers that store users’ private keys on the servers. It is crucial to acknowledge that no single method can offer comprehensive protection against fraudulent schemes. While S/MIMEs provide the confidentiality and integrity of contents, they are considered weak against VEC attacks. Consequently, it is advisable to employ a combination of security measures, including TLS, S/MIMEs, and the suggested method, to fortify a company’s defense against such attacks [24];
(7) **Digital signatures**: Digital signatures in email communication are instrumental in fortifying the security and reliability of electronic exchanges. These signatures, generated through cryptographic algorithms, assure the authenticity of the sender and the integrity of the message content. This form of security prevents unauthorized access and tampering during transmission, offering a vital defense against cyber threats. Moreover, digital signatures provide a crucial element of non-repudiation, making it challenging for senders to deny their involvement in a specific message. This not only enhances accountability but also minimizes the risk of disputes over message origins. In an environment where sensitive information is regularly shared, the adoption of digital signatures instills confidence, establishing a secure foundation for electronic communication [45,46]. Digital signatures, while offering significant advantages, are not without their drawbacks. A notable vulnerability is the issue of “unobservability” in electronic documents. This means that, in certain cases, the content of a digitally signed document may be concealed or difficult to discern. According to Lax et al. [47], unlike traditional documents that can be interpreted by humans through direct observation, digital documents rely on machine-level interpretation and require complex instruments such as computers for viewing and signing. This inherent complexity introduces vulnerabilities, particularly in ensuring the consistency and reliability of these instruments. The unobservability of digital documents poses a challenge to the direct link between the signature and the information’s integrity, making it inherently weaker compared to handwritten signatures. Despite technical measures addressing bit-level modifications, concerns persist regarding the reliability of the instruments used for viewing and signing documents, rendering digital signatures inherently weak. The paper highlights various vulnerabilities resulting from this unobservability and explores potential solutions, emphasizing the balance between security and usability in the context of digital signatures. Malicious actors can exploit disadvantages like the abovementioned to their advantage, compromising the transparency and verifiability that digital signatures aim to provide. To address this, there is a critical need for secure and efficient verification methods. Advanced algorithms can play a pivotal role in enhancing the verification process, ensuring that the integrity and authenticity of digitally signed documents are upheld [48]. Implementing sophisticated algorithms can mitigate the risks associated with unobservability, making it more challenging for malicious actors to manipulate or conceal electronic content.

#### 3.3.2. Non-Technical Methods

1. **Employee training**: Ongoing employee training is vital to empower staff in identifying, reporting, and handling BEC attacks effectively. It is especially crucial to provide regular training to sensitive sectors, like the financial department, focusing on social engineering techniques and BEC schemes. Employees should also exercise caution when dealing with hyperlinks, attachments, name misspellings, sudden wire transfer requests, or altered account details. Encouraging the verification of vendor information is equally essential and strongly recommended. It is important to recognize that social engineering and BEC schemes are continuously evolving, underscoring the necessity for continuous and up-to-date training sessions. By remaining vigilant and well informed, employees can play a crucial role in safeguarding the organization against threats like BEC attacks [49,50];
2. **Social engineering departments**: Creating a dedicated social engineering department is essential when it comes to large companies. This department should consist of employees who have undergone specialized training in social engineering and open-source intelligence (OSINT) investigations. Leveraging OSINT tools, they can conduct thorough investigations of high-profile targets within the company to identify potential data breaches and leaks. Utilizing free online services like Have I Been Pwned [51] and DeHashed [52], they can assess vulnerabilities and gather crucial information to safeguard against BEC attempts. Recognizing that the information gathered could be exploited by malicious attackers in order to make the profile of a target, understanding the existing gaps and potential compromises in the company’s profile becomes crucial. By proactively identifying and addressing these weaknesses, the organization can effectively prevent and detect future BEC attacks, fortifying its cybersecurity defenses [53];
3. **Defining policies**: To bolster security measures, the implementation of a set of comprehensive policies and internal guidelines that prioritize safeguarding information sharing and financial transactions is needed. By defining and adhering to these policies, the organization can significantly mitigate the risks associated with BEC attacks and enhance their overall cybersecurity. Characteristic examples of these policies are as follows:
  - Prohibition of the use of email requests for fund transfers and, instead, mandating the presence of multiple individuals or at least a vocal confirmation for financial transactions;
  - Strong communication protocols for phone-based interactions by enforcing identity verification questions to prevent unauthorized data disclosure;
  - Encouragement of the swift reporting of any security incidents to enable quicker action and resolution;
  - Endorsement of strong password policies.

When it comes to safeguarding against business email compromise (BEC) attacks, there are a variety of protective measures, each with its own set of advantages and drawbacks, as discussed earlier.

DMARC, for instance, is a potent tool that serves to shield against domain spoofing, ensuring the integrity and authenticity of emails through the use of digital signatures in outgoing messages. However, determined attackers armed with lookalike domains or adept social engineering techniques can bypass this defense mechanism by manipulating email addresses, aiming to deceive recipients.

Antivirus and antimalware software plays a crucial role in guarding users against malicious URLs and programs that exploit system vulnerabilities. Nevertheless, its effectiveness relies heavily on widespread adoption and regular updates, and it remains susceptible to emerging threats like zero-day exploits. Furthermore, it is ill equipped in countering social engineering techniques.

Machine learning algorithms offer promise in classifying emails and raising the awareness of potential red flags by scrutinizing data and identifying patterns associated with BEC attacks. However, to function efficiently, machine learning requires the analysis of large amounts of data, particularly within email body text.

Encryption, as previously noted, provides substantial security benefits. Still, it is not without its share of challenges and disadvantages, including complexity in its implementation, the need for diligent key management, compatibility issues across various platforms, and the added processing overhead it demands.

Multi-factor authentication (MFA), a widely endorsed security practice, furnishes an additional layer of protection for user accounts. Nonetheless, it introduces its own set of challenges. Users may find MFA less convenient, particularly during the setup phase. Compatibility issues may arise, especially when dealing with diverse systems and applications. Moreover, MFA does not eliminate the risk of phishing attacks, especially if users are not adequately educated about its usage and potential vulnerabilities. Additionally, when it comes to BEC attacks, recipients have no way of discerning whether the sender employed MFA, leaving room for uncertainty.

Finally, non-technical strategies for guarding against BEC attacks, such as employee training initiatives, policy formulation, and the establishment of specialized departments, like those focusing on social engineering, offer valuable layers of defense. However, they are contingent on continuous education and policy updates, making them susceptible to the ever-evolving nature of BEC attacks. These approaches also rely heavily on human factors, which introduce their own unique challenges. In essence, while these non-technical strategies are valuable components of a comprehensive BEC defense strategy, they require constant vigilance and adaptation. Cybercriminals continually refine their tactics, which necessitates ongoing education and policy refinement. Moreover, they demand a deep understanding of the human element in security, acknowledging that the human factor can both bolster and undermine the effectiveness of these defenses.

## 4. Proposed Methodology

To implement our solution, the BEC Defender collects the sender’s MAC address, IP address, and hostname, and a timestamp. Each of these components serves a specific purpose: the MAC address acts as an identifier, challenging spoofing attempts; the IP address and username provide additional data for forensic analysis and database classification; and the timestamp ensures that the validation token remains valid for a limited duration, typically three hours from its creation.

In the next step, a message authentication code (MAC) is generated using the MAC address, and all the above mentioned data are converted into a QR code, which can be sent along with the email. When the receiver receives the email, they can decode the QR code using the BEC Defender and then proceed to the sender verification procedure.

This authentication is accomplished by cross-referencing the sender against a preapproved list and comparing the original timestamp with the recipient’s system time. The time differential between the two timestamps should not exceed 10,800 s, which is equivalent to a 3 h window, effectively preventing potential attacks, such as man-in-the-middle exploits and replay attacks.

### 4.1. Background

This Section presents the concepts necessary for presenting the subsequent Sections and the proposed methodology. The proposed solution builds on several well-established cryptographic techniques, including message authentication code (MAC) and QR code technology.

#### 4.1.1. Message Authentication Code (MAC)

A message authentication code (MAC) is a cryptographic code that is enclosed in a message or in data in order to ensure the message’s integrity and authenticity. Message authentication codes (MACs) offer an effective method for verifying that a message remains unaltered during transmission and that the sender’s identity is genuine [54]. The generation of a message authentication code involves applying a specific cryptographic algorithm to the message, resulting in the creation of a unique code. This process ensures the integrity and authenticity of the message. Upon receiving the message and the associated MAC, the recipient has the ability to recompose the MAC using the same key and the received message. If the recalculated MAC matches the one initially received, the receiver confirms that the message remained unaltered during transmission and indeed originated from the party possessing the corresponding secret key.

MACs are extensively used in a variety of security protocols and applications, like network communication, secure data storage, banking applications, and validation techniques. They offer a reliable way to confirm the authenticity and integrity of data, thereby establishing themselves as an element of secure communication [55,56].

#### 4.1.2. Quick-Response (QR) Codes

Quick-Response (QR) codes [57] are two-dimensional barcodes that can store information such as text, URLs, or contact information. QR codes are widely used in various applications, such as ticketing, inventory management, and marketing. The advantage of QR codes is their ability to store a large amount of data, making them ideal for transmitting encrypted data securely. They are also considered a user-friendly approach when it comes to transferring data.

### 4.2. Implementation

In this Section, we present the development of the algorithm designed to fortify online communications against malicious acts, with a specific focus on countering business email compromise (BEC) attacks. The algorithm was created using the Python programming language and was tested across both the Windows and Linux operating systems. For the evaluation process, testing emails were established using Gmail accounts, serving as the sender and receiver in simulated scenarios.

By employing Python as the programming language, the algorithm leverages its versatility and functionality, ensuring robust implementation and ease of integration. The choice of the Windows and Linux OS terminals allows for broader applicability and cross-platform compatibility, catering to a wide range of users.

The use of Gmail accounts as the testing environment enables real-world simulations, facilitating the comprehensive testing of the algorithm’s effectiveness in safeguarding against BEC attacks. By mimicking actual communication scenarios, the algorithm’s resilience can be thoroughly assessed.

Throughout this Section, we will delve into the core concepts, methodologies, and step-by-step creation of the algorithm, elucidating how it bolsters online communication security and acts as a crucial defense against malicious cyber threats, particularly in the context of BEC attacks.

**Figure 3:**
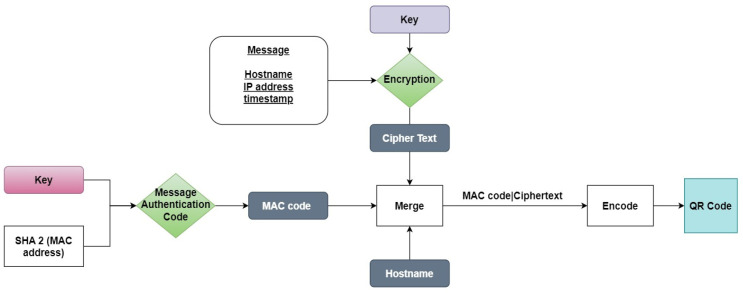
Description of BEC-Defender algorithm for sender procedure.

**Figure 4:**
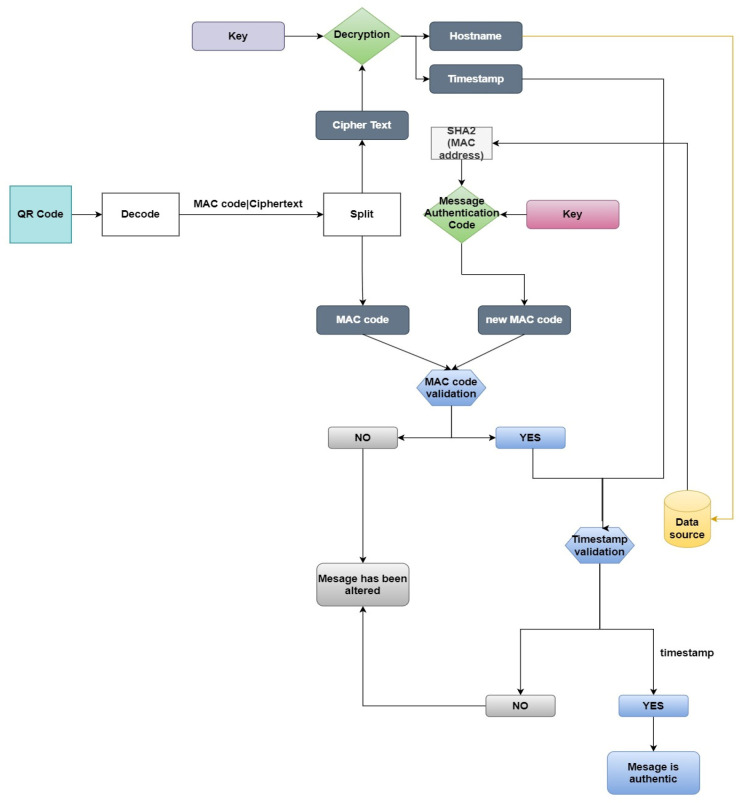
Description of BEC-Defender algorithm for receiver procedure.

Figure 3 and Figure 4 describe the procedure of the QR code generation, which contains the sender’s information and the procedure used for the decryption and evaluation of the sender identity through a comparison of the information included. The algorithm aims to serve as a robust security measure for email communication.

#### Initialization Phase

Our scheme incorporates a crucial initialization phase to ensure secure communication. In the case of communication between different enterprises/organizations, it is suggested that both recipients are pre-distributed a common symmetric key via physical interaction or by exchanging post office mail. This ensures the confidentiality and integrity of the key exchange process. Once this prerequisite is met, the recipients can proceed to register their MAC addresses by following the algorithm outlined below.

**MAC address Database creation**

Each user has to register their MAC address via one recipient. The user has to send a registration form to the technical department that contains the following information:

1. SHA2 MAC address;
2. Hostname (hostnames are typically configured by users for their devices, particularly in the case of desktops and laptops).

This information is stored in a database in the user’s private system and contains two columns of information with the above gathered data. Each recipient follows the following algorithm to register their MAC address:

Algorithm for Registration

Input: MAC Address, Hostname

Output: Success or Failure

1. Generate the Hash Code for the MAC Address using the SHA2 algorithm and store it in SHA2MAC.
2. If SHA2MAC Address exists in Database, then
  - Write “Already Exists”
  - Returns “Failure”
3. else
  - Store SHA2MAC and hostname and new record generated in the database
  - Write “Record Saved”
  - Returns “Success”
4. end if
5. Stop.

**Sender**

From the sender’s perspective, this algorithm will execute a series of well-defined steps to guarantee the secure transmission of information. More analytically,

1. MAC address collection and the hashing of the MAC address: The algorithm encodes the sender’s MAC address using a keyed-hash message authentication code (HMAC). We utilize the SHA2 hash function to generate the message authentication code, employing a shared symmetric key;
2. Cipher text creation and encryption: Next, the algorithm encrypts the information of the IP address, hostname, and timestamp. The message is encrypted using a 32-byte key, resulting in a ciphertext. More specifically, Fernet encryption is employed with the help of the equivalent library in Python;
3. Cipher text and MAC code integration: The cipher text and message authentication code are merged into a new message structure. This process is facilitated by the use of the “|” character, which serves as a delimiter;
4. QR code generation: In the final step of the process, the MAC code, along with the cipher text, are encoded into a QR code. This QR code can be conveniently included in the sender’s email, making it a convenient and secure method for transmitting the data.

The proposed scheme for secure communication is based on symmetric cryptography. In order to enhance the strength of the communication and mitigate the risk of relying solely on a single symmetric key, we have opted for the utilization of two common secret keys. This approach safeguards against potential vulnerabilities that may arise from phishing attacks, during which the key could be exposed. By employing this dual-key system, we ensure a higher level of security and resilience in our communication framework. To enhance the security and privacy of our communication system, we have implemented a robust scheme that employs two distinct secret keys. The first key is exclusively used for encryption, ensuring that the transmitted data remain confidential and protected from unauthorized access. The second key serves the crucial purpose of generating and verifying the message authentication code (MAC), guaranteeing the authenticity and integrity of the exchanged messages.

**Receiver**

Upon receiving the encrypted QR code to the testing email, the algorithm commences the validation process:

1. First, it decodes the QR code and, with the use of the “|” character, extracts the cipher text and the message authentication code;
2. Next, the algorithm commences the decryption of the cipher text using the encryption key in order to extract the hostname and the timestamp;
3. In the initial validation process, the receiver reconstructs the message authentication code (MAC) using the authentication key and the stored hashed value of the MAC address of the data source linked to the corresponding hostname. This reconstructed MAC code is then compared with the received MAC code. If the two MAC codes match, the algorithm proceeds to the next validation step. However, if the MAC codes do not match, the algorithm generates the message “Message has been altered”;
4. Finally, the next validation requirement involves assessing the decrypted timestamp. By comparing the sender’s timestamp with the receiver’s timestamp (the blue line in Figure 3), the algorithm ensures that the timeframe between the QR code’s creation and receipt is within three hours. If this condition is met, the algorithm confirms the third validation requirement and prints the message “Message is authentic”. However, if the timeframe exceeds three hours, indicating a potential replay attack or unauthorized delay, the algorithm identifies it as a red flag.

In conclusion, the BEC-Defender algorithm introduces a robust validation system to guarantee the authenticity of the sender’s identity while eradicating the risks of replay attacks and man-in-the-middle exploits. Through the utilization of data encryption, MAC code verification, the creation of QR codes, and the incorporation of timestamps as distinctive markers, the BEC-Defender algorithm delivers a secure and effective strategy for fortifying email communications against potential threats and serves as a proactive defense against BEC attacks.

## 5. Performance Evaluation

To thoroughly evaluate the robustness of the proposed algorithm and its efficacy in enhancing email communication security, an extensive testing phase was conducted. This comprehensive assessment encompassed 10 diverse email accounts sourced from various providers, such as Gmail, Yahoo, and Proton Mail. To ensure comprehensive testing, virtual machines were utilized to simulate different operating systems, including Windows 10, Ubuntu, and Kali Linux.

Over the course of the evaluation, more than 100 tests were carried out on these virtual machines, with each test involving the use of different MAC addresses, while the predefined list of MAC addresses in the algorithm databases was limited to 10 permittable values. To achieve this, the Technitium MAC Address Changer v6 program was employed on Windows OS, while the MacChanger program was utilized on the Ubuntu/Kali Linux operating systems. These tools facilitated the generation of distinct MAC addresses for each test by spoofing the original MAC address, enabling a wide range of scenarios for examination.

By conducting tests across various email providers and operating systems, the algorithm’s adaptability and efficiency were thoroughly examined. The use of virtual machines ensured a controlled testing environment, eliminating potential interference from actual hardware configurations.

Table A1 (Appendix A–Table A1) shows the permitted ten out of fifty MAC addresses for the tests conducted in the Windows operating system with various spoofed MAC addresses, which are presented in Table A2 (Appendix A–Table A2).

To assess the viability of the proposed solution, we consider a scenario in which Sender (A) aims to transmit an email to Receiver (B) containing sensitive information or orders. It is essential for Sender (A) to guarantee that the recipient can authenticate their identity securely. Sender (A) incorporates the QR code into the email body or attaches it. The QR code that Sender (A) created though the BEC-Defender algorithm encloses the encrypted data and the message authentication code, as mentioned earlier in the Proposed Methodology Section.

Upon receipt, Receiver (B) employs an algorithm to decode the QR code and verify the identity of Sender (A). The algorithm performs a series of cross-validations:

1. The algorithm cross-references the sender’s MAC code with the generated MAC code. If the two MAC codes are identical, the algorithm proceeds to the next validation. Through this procedure, the algorithm verifies whether the encoded MAC address included in the QR code matches any of the preapproved values on the data source. If a match is found, the algorithm proceeds to the third and final validation;

2. In the final step, the algorithm compares the timestamp of the QR code creation with the current execution timestamp. If the time elapsed between these two events is less than three hours, the algorithm concludes all the validations and prints out a “Message is authentic” response.

If any of the above validations fail, the algorithm will generate a response indicating that the message has been altered. To enhance the resilience of the algorithm against replay attacks, we can introduce a random number into the calculation of the SHA2 hash function.

As a result, Receiver (B) will have two validation procedures to verify the identity of Sender (A). The first validation ensures that the email originated from a trusted source. The second validation, tied to the timeframe, protects against the potential interception and misuse of the unique QR code by malicious actors attempting to create fraudulent emails with identical tokens. This multi-layered authentication process significantly enhances the security and trustworthiness of email communication, particularly in scenarios that require stringent identity validation.

Table 2 exhibits some of the results during the testing phase. The first column of Table 2 refers to 10 of the total MAC address values (Appendix A Table A1 and Table A2).

The second column of Table 2 refers to the first validation process of the BEC-Defender algorithm, which, as described earlier in the Proposed Methodology Section, compares the new generated MAC commenced by the algorithm for the receiver with that of the sender.

Five out of ten of these values are permitted, meaning that they are part of the database that includes permitted MAC address values and which the algorithm uses to cross-reference with the MAC address included in the sender’s QR code. The remaining five MAC addresses are those not included in the database, meaning that they will not pass the validation process.

Columns four, five, and six in Table 2 correspond to the creation date/time of the QR code, the validation date/time of the QR code, and the timestamp validation status. In order to characterize the timestamp validation status as valid, it is required that the duration between the creation and validation date/time of the QR code falls within the 3 h timeframe.

Through these various tests, the proposed algorithm’s solution was tested, providing valuable insights into its effectiveness at safeguarding email communication against potential threats, like business email compromise (BEC) attacks. The results of these tests play a vital role in validating the algorithm’s capabilities and establishing its credibility as a powerful solution in the realm of cyber defense.

## 6. Discussion

### 6.1. Advantages–Limitations

The proposed solution offers several advantages over traditional email systems. Firstly, the use of QR codes as an additional layer of authentication enhances the security of the email communication. By requiring the sender to provide additional identification data encrypted within the QR code, the system mitigates the risk of impersonation and prevents unauthorized individuals from sending fraudulent emails.

Secondly, the encryption of the sender’s data provides a high level of confidentiality and data integrity. The use of encryption ensures that only the intended recipient can decrypt and access the sender’s information, preventing eavesdropping and unauthorized access.

Furthermore, the incorporation of a preapproved list of MAC addresses adds an extra layer of security. By comparing the sender’s information with this list, the program can quickly identify and flag any suspicious or unauthorized senders, reducing the risk of falling victim to BEC attacks. The MAC address value of the sender is encoded in order to prevent potential data theft via eavesdropping.

Ultimately, by including a timestamp in the message and implementing a three-hour window, the algorithm aims to protect against replay attacks and potential man-in-the-middle exploits.

Despite its advantages, the proposed solution also has some limitations that need to be considered. Firstly, the program relies on both the sender and receiver using the system for secure email communication. This means that the widespread adoption and awareness of the program in a company are essential for its effectiveness. Additionally, the initial setup and configuration process may require technical expertise, potentially limiting its accessibility to only IT staff.

Secondly, the program’s reliance on the preapproved list assumes that the list itself is secure and free from any unauthorized modifications. Regular reviews and updates of the approved list are necessary to maintain its integrity.

### 6.2. Conclusion–Future Work

In conclusion, email security is a critical concern in today’s digital landscape, with BEC attacks posing a significant threat. The BEC Defender is a promising methodology that uses a programmable QR code system for secure email communication, incorporates encryption and authentication techniques, and shows promise in enhancing email security and preventing BEC attacks.

By leveraging MAC code, data encryption, and QR code technology and a preapproved list of senders’ information, the BEC Defender adds layers of confidentiality, integrity, and authentication to email communication. However, it is essential to address the limitations, such as user adoption, the system setup, and the need for continuous updates to maintain its effectiveness.

Future research directions may involve the use of other types of encryptions, updating and refining the user interface to make it more user-friendly and accessible, exploring the integration of additional authentication methods, such as two-factor authentication or biometric authentication, and conducting thorough security audits to identify and address potential vulnerabilities. Moreover, future work could aim to adapt our BEC-Defender algorithm solution into an add-on or plugin that is compatible with a range of web browsers, simplifying its usage for the end user.

With continuous improvements and widespread adoption, the proposed methodology has the potential to significantly enhance email security, protect against BEC attacks, and provide users with a safer and more secure email communication experience.

## Figures and Tables

**Table 1:** Literature review related to QR technology in document/data or user authentication.

Journal	Summary
[7]**,** **Degree certificate authentication using QR code and smartphone,** ***International Journal of Computer Applications*** **, 2015**	Authentication of university degree with the use of QR code.
[8]**,** **Embedding a blockchain technology pattern into the QR code, for an authentication certificate** ***Jurnal Online Informatika*** **, 2020**	Authentication of university degree with the use of QR code.
[9]**,** **Paper-based document authentication using digital signature and qr code** ***International Conference on Computer Engineering and Technology, 2012***	Document verification with the use of QR code.
[10]**,** **Two-level QR code for private message sharing and document authentication** ***IEEE Transactions on Information Forensics and Security*** **, 2015**	Document verification with the use of QR code.
[11]**,** **Elliptic curve cryptography** **Ubiquity, 2008**	This paper describes the Elliptic Curve Cryptography algorithm and its suitability for smart cards.
[13]**,** **Multiple schemes for mobile payment authentication using QR code and visual cryptography** ***Mobile Information Systems*** **, 2017**	This paper proposes a methodology for mobile payment authentication that combines visual cryptography (VCS) and aesthetic QR codes.
[14]**,** **A novel user authentication scheme based on QR-code** ***Journal of Networks*** **, 2010**	This paper proposes a QR code-based, one-time-password authentication protocol, which the author claims eliminates the usage of the password verification table in an improved, cost-effective way.
[15]**,** **A Study on Authentication System Using QR Code for Mobile Cloud Computing Environment** **In *Future Information Technology*. *Communications in Computer and Information Science*, Park, J.J. Yang, L.T. Lee, C. Eds. Springer, Berlin, Heidelberg, 2011**	This paper proposes an authentication system that optimizes network traffic usage in mobile cloud environments by implementing QR codes.
[16]**,** **A mobile based anti-phishing authentication scheme using QR code** **International Conference on Mobile IT Convergence IEEE, September, 2011**	This paper proposes a QR-based, anti-phishing authentication scheme that is secure against phishing attacks.
[17]**,** **Mutual authentication of nodes using session token with fingerprint and MAC address validation** ***Egyptian Informatics Journal*** **, 2021**	This paper proposes an algorithm for message and data transfer using an authentication token containing six-digit random numbers with the SHA-hash parts of the sender’s and receiver’s MAC addresses.
[18]**,** **An overview of MANET: History, challenges and applications.** ***Indian Journal of Computer Science and Engineering*** **(IJCSE), 2012**	This paper proposes an algorithm especially for Mobile Ad Hoc Networks (MANETs).
[19]**,** **QR Code Authentication with Embedded Message Authentication Code** ***Mobile Networks and Applications*** **, 2017**	This paper proposes a QR code authentication method that includes hidden authentication elements like message authentication codes and cryptographic signatures.

**Table 2:** Results of various tests using BEC-Defender algorithm. The algorithm performs various validations to ensure the sender’s verification.

MAC Address	MAC Code Validation	MAC Address Validation Status	QR Creation Date/Time	QR Validation Date/Time	Timestamp Validation Status
02-F2-74-7F-8F-5A	Valid	Valid	10 July 2023	10 July 2023	Valid
			11:05:23	12:19:23	
02-3D-08-AB-95-56	Invalid	Invalid	11 July 2023	11 July 2023	Valid
			09:14:18	11:22:06	
18-97-FF-0B-42-FB	Valid	Valid	14 July 2023	14 July 2023	Invalid
			14:08:44	17:10:59	
00-20-74-35-4D-29	Invalid	Invalid	14 July 2023	14 July 2023	Valid
			18:23:39	19:05:12	
00-25-16-7B-9F-FC	Invalid	Invalid	22 July 2023	22 July 2023	Invalid
			08:42:17	14:01:28	
00-11-0A-7E-5A-AC	Valid	Valid	29 July 2023	29 July 2023	Invalid
			18:52:24	22:06:42	
54-4A-00-05-3B-CC	Invalid	Invalid	4 August 2023	4 August 2023	Valid
			10:08:36	10:54:49	
00-05-08-A5-E3-C7	Valid	Valid	8 August 2023	8 August 2023	Valid
			09:00:23	11:59:04	
18-14-20-6E-30-E1	Valid	Valid	8 August 2023	8 August 2023	Invalid
			09:26:11	15:29:57	
F4-15-63-C8-63-11	Invalid	Invalid	11 August 2023	11 August 2023	Valid
			12:27:18	14:04:36	

## Data Availability

Data are contained within the article.

## Appendix A

**Table A1:** Permitted MAC addresses.

Permitted MAC Addresses
02-F2-74-7F-8F-5A
00-05-54-05-63-14
18-97-FF-0B-42-FB
50-F0-D3-3B-A8-B8
8C-7C-92-51-42-E6
34-A7-09-D2-12-96
00-11-0A-7E-5A-AC
00-15-2A-CF-6D-67
00-05-08-A5-E3-C7
18-14-20-6E-30-E1

**Table A2:** Non-permitted MAC addresses.

Non-Permitted MAC Addresses	
02-70-B0-A7-24-A2	00-07-A9-FA-63-A0
02-3D-08-AB-95-56	00-25-B9-6D-80-DE
02-BD-D1-75-55-D1	00-30-AC-DB-7C-24
02-1A-2C-E6-D4-9B	24-93-CA-D6-85-5F
02-02-A7-57-B4-A8	44-6D-57-D8-51-73
02-00-85-4E-FD-EA	00-19-49-FA-02-0A
02-01-C6-9D-2D-AD	00-04-27-DC-BC-1C
02-0E-F0-92-A5-1D	54-4A-00-05-3B-CC
02-68-95-E8-13-13	00-0E-D0-EB-D0-68
00-20-74-35-4D-29	00-18-FA-C6-0A-3E
00-C0-AD-10-23-13	00-1A-6A-10-57-AD
02-1E-43-C7-B8-FD	00-16-03-24-7C-DD
00-25-16-7B-9F-FC	00-A0-B5-46-26-55
00-11-D8-6A-C8-18	C0-5E-6F-43-14-B0
00-0D-C1-08-EC-26	00-0A-DF-41-FC-4B
00-1A-94-82-42-D1	00-25-D8-E0-ED-54
00-0A-06-96-27-E4	94-8B-03-7B-84-1D
00-90-78-3C-78-86	F4-15-63-C8-63-11
00-01-42-F2-C5-7B	00-1F-75-66-3D-9A
00-30-71-87-34-1D	64-9A-BE-47-97-8F
